# Nutritional Value, Mineral Composition, Secondary Metabolites, and Antioxidant Activity of Some Wild Geophyte Sedges and Grasses

**DOI:** 10.3390/plants8120569

**Published:** 2019-12-04

**Authors:** Saud L. Al-Rowaily, Ahmed M. Abd-ElGawad, Suliman M. Alghanem, Wafa’a A. Al-Taisan, Yasser A. El-Amier

**Affiliations:** 1 Plant Production Department, College of Food & Agriculture Sciences, King Saud University, P.O. Box 2460, Riyadh 11451, Saudi Arabia; srowaily@ksu.edu.sa; 2 Department of Botany, Faculty of Science, Mansoura University, Mansoura 35516, Egypt; yasran@mans.edu.eg; 3 Biology Department, Faculty of Science, Tabuk University, Tabuk 71491, Saudi Arabia; s-alghanem@ut.edu.sa; 4 Department of Biology, College of Science, Imam Abdulrahman Bin Faisal University, P.O. Box 1982, Dammam 31441, Saudi Arabia; waltaisan@iau.edu.sa

**Keywords:** *Cyperus*, grasses, nutritive value, forage, minerals, drought, medicinal plants

## Abstract

Geophytes are plants with underground storage organs including bulbs, corms, tubers, and rhizomes, often physiologically active and able to survive during harsh environmental conditions. This study is conducted to assess the nutritive value, mineral composition, bioactive metabolites, and antioxidant activity of five wild geophytes (*Cyperus capitatus*, *C. conglomeratus*, *Elymus farctus, Lasiurus scindicus,* and *Panicum turgidum*) collected from the Nile Delta coast and inland desert. The proximate composition including dry matter, moisture content, ash content, fiber, fat, protein, sucrose, and glucose were determined. Also, total carbohydrates, total digestible nutrients (TDN), and nutritive values were calculated. Macro- and micro-minerals were also determined in the studied geophytes. Total phenolics, total flavonoids, alkaloids, saponins, and tannins were determined. Antioxidant activity was evaluated based on 2,2-diphenyl-1-picrylhydrazyl (DPPH) radicle scavenging. Based on the nutritive value, the studied geophytes are ranked as follows: *E. farctus* > *C. conglomeratus* > *L. scindicus* > *P. turgidum > C. capitatus*. The mineral analysis reveals a sufficient amount of macro- and micro-elements in the studied geophytes while the microelements levels in the studied wild plants exist as Fe > Mn > Zn > Cu. *Cyperus conglomeratus* attained the highest concentrations of all determined secondary metabolites. On the other hand, *C. conglomeratus*, *C. capitatus*, and *P. turgidum* extracts showed strong scavenging activity (EC_50_ < 1 mg mL^−1^), while extracts of *E. farctus* and *L. scindicus* exhibited moderate scavenging activity (1 ≤ EC_50_ ≤ 2 mg mL^−1^). The present data reveal that geophytes under investigation could be used as good forage plants, especially in arid habitats. In addition, *C. conglomeratus* could be a potentially important candidate for natural antioxidants as it attained high contents of the bioactive constituents.

## 1. Introduction

In developing countries, the high population growth rates combined with limited and rapidly decreasing land for food and forage production have led to the need to intensify agricultural production to bridge the gap between food needs and supplies as well as to ensure appropriate human nutrition. Intensification, in the ruminant production systems, implies extending the feed sustainable resource base to compensate for the deficiencies of rangeland and natural grasslands, moreover increasing the low quality and seasonal nature of feed resources [1].

Geophytes are plants with underground storage organs that include bulbs, corms, tubers, and rhizomes, which appear to be promising raw materials for various economic uses [2]. Geophytes are often physiologically active even when they lack leaves; they can survive during harsh environmental conditions as they reserve carbohydrates and water until the favorable conditions come again. There are no evergreen plants that are geophytes in natural habitats, whereas geophytes are those vascular plants that survive unfavorable conditions for growth [3]. These geophytes have high diversity in the Mediterranean-type ecosystems, where they are considered as most common in seasonal climates [4].

Forages are plants or parts of plants eaten by domesticated animals and wildlife. Herbs (Poaceae family) are normally herbaceous, which indicate that they produce a seed, do not create woody tissues, and die at the end of a growing season [5]. The economic importance of grasses lies in their role as an important food source, which produces edible grain, the bulk of which, provides a rich source of carbohydrates, protein, oil, and some vitamins [6]. In addition, grasses have adapted to the full range of environmental extremes from the coldest regions and highest elevations, and from fully aquatic habitats to deserts. On the other hand, plants of the genus *Cyperus* or sedges (Cyperaceae family) include some common wild species found in the desert habitat. Some types of this family are used in traditional folk medicine and as food, as well as the vegetative yield used as forage for domestic and wild animals [7].

Therefore, communities in arid regions have a long history of using sustainable renewable resources for uncultivated areas to produce more food for people, animal feed, and the manufacture of our local raw materials [8,9]. Mashaly et al. [10] studied the nutritive value of some members of Cyperaceae (*Bolboschoenus glaucus*, *Cyperus laevigatus*, *Schoenoplectus litoralis*) and Poaceae (*Echinochloa stagnina, Leptochloa fusca*, *Panicum repens*, *Paspalidium geminatum*, *Schoenoplectus litoralis*, and *Sorghum virgatum*). Heneidy and Halmy [11] reported that *Panicum turgidum* has high nutritive value and is considered a good forage plant.

This study is conducted to assess the nutritional value, mineral composition, secondary metabolites, and antioxidant activity of five wild-grown geophytes; two of Cyperaceae (*Cyperus capitatus* and *C. conglomeratus*) and three of Poaceae (*Elymus farctus*, *Lasiurus scindicus*, and *Panicum turgidum*) collected from the Egyptian coastal desert (Deltaic Mediterranean coast) and inland desert (Wadi Hagoul).

## 2. Results and Discussion

### 2.1. Proximate Composition

Dry matter, moisture, ash, and fiber contents of the five geophytes showed non-significant differences between the studied geophytes (Table 1). The value difference was not significant among the studied plant species. Dry matter content is the actual amount of nutrients that leave water, acids, and bases, if any. In the present study, the highest (91.67%) dry matter content was determined in *C. conglomeratus,* and the lowest (87.10%) value was obtained from *C. capitatus*. Data obtained from this study are in harmony with other reported wild species such as *Cyperus laevigatus*, *Bolboschoenus glaucus*, *Panicum repens*, and *Sorghum virgatum* [10]. The dry matter content of the plants varies according to various factors such as plant species, parts of the plant, growing conditions, soil and environment [12]. In our view, the high content of dry matter in the studied plants can be ascribed to the fact that these plants grow naturally in sandy habitats with low content of water.

The moisture content of plants, ranging from 8.33% in *C. conglomeratus* to 12.90% in *C. capitatus*, makes them more stable during storage and packaging. The ash content oscillated between 8.62% for *P. turgidum* and 10.75% for *C. capitatus*. On the other hand, there was a slight variation between the crude fibers of the studied plant (Table 1). The highest (13.84%) and lowest (11.97%) crude fiber were from *P. turgidum* and *C. conglomeratus*, respectively. The range of the crude fibers in the studied plants are in harmony with that reported for *C. rotundus* [13], *C. laevigatus*, and *P. geminatum* [10], while it was lower than those reported in *B. glaucus*, *S. litoralis*, *E. stagnina, L. fusca*, *P. repens*, and *S. virgatum* [10]. These variations may probably reflect the difference in origin, season of use (wet season versus dry season), plant species, and growth phase [14].

The total protein content of feed plants is considered as an indicator of the nutritional value for ruminants. In the present study, the protein content of the studied geophytes was relatively high in general (>10.0%) compared with other grasses (3.0–10.0%) reported by El-Amier and Abdullah [15], Abdou Bouba et al. [14], and Imam et al. [16]. The crude protein contents showed a significant variation (*p* = 0.0013) among the investigated plants, and it was varied between 12.06% (*P. turgidum*) to 17.13% (*C. conglomeratus*). The wild edible plants such as *Malva neglecta* and *Polygonum bistorda* had 2.35% and 11.56% protein contents, respectively [17,18]. In the cultivated forage such as *Medicago sativa*, *Trifolium alexandrinum*, *E. stagnina*, and *Cynodon dactylon*, protein contents were 19.70%, 17.19%, 16.38%, and 12.63%, respectively [19,20]. The content of protein in the present study was comparable to those reported for *E. stagnina* and *L. fusca*, while it was higher than those reported in *C. rotundus*, *C. laevigatus*, *P. geminatum*, *B. glaucus*, *S. litoralis*, *P. repens*, *S. virgatum* [10], and *P. turgidum* [11].

Although fats are a concentrated source of energy, they do not constitute a major source of energy from forages [21]. The fat contents of the studied plant revealed a highly significant variation (*p* = 0.0004). The highest value of lipids (5.87%) was recorded in *P. turgidum,* and the lowest value (3.19%) was that of *C. conglomeratus* (Table 1). This is in agreement with the results of other reports [22,23], but it is higher than those reported by Zahran and El-Amier [1]. However, lower contents of lipids were reported in the other members of Cyperaceae and Poaceae [10] compared to the present results.

Regarding the contents of glucose and sucrose, comparable amounts were determined among the studied geophytes (Table 1). The highest concentrations of glucose (0.94%) and sucrose (1.98%) were determined in *C. conglomeratus*. It was reported that the contents of glucose and sucrose in *C. laevigatus* were 0.34% and 4.74%, respectively [10]. 

**Figure 1:**
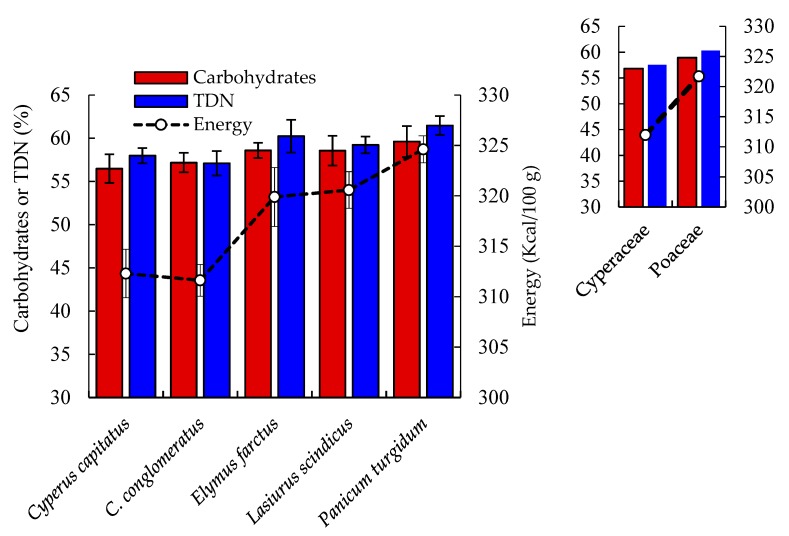
Carbohydrates, total digestible nutrients (TDN), and energy of the selected geophyte plants in the Egyptian desert. Values are mean (*n* = 3) with standard error bars.

In any forage plants, the nutritional status depends upon the contents of protein, lipids, and carbohydrates. However, the palatability and digestibility of each plant depend on the composition of these organic nutrients, as well as minerals, vitamins, and antinutritional compounds [24]. Some calculated parameters, including total carbohydrates, energy, and total digestible nutrients (TDNs), were determined for the five studied geophytes. Slight variations in both carbohydrates and TDNs were observed among the studied plants (Figure 1). Carbohydrate value ranged from 56.48% in *C. capitatus* to 59.61% in *P. turgidum*. The dry matter of forage crops should contain about 50–80% carbohydrates, while if this ratio is too low, then supplements can be added [24]. The average carbohydrate contents of Cyperaceae and Poaceae were comparable.

*Elymus farctus* showed the highest energy level at 324.61 kcal 100 g^−1^ DW, while *C. capitatus* attained the lowest level (311.62 kcal 100 g^−1^). Our data on studied geophytes are in agreement with that reported for other species of Cyperaceae (*C. laevigatus*, *B. glaucus*, *S. litoralis*,) and Poaceae (*P. geminatum*, *P. repens*, *E. stagnina*, and *L. fusca*), while it was lower than the metabolizable energy of *S. virgatum* (294.56 kcal 100 g^−1^ DW). On the other hand, the TDNs are used to describe energy available in food to animals after digestion losses have been deduced. The literature data on the annual mean of the TDN value showed a figure of 75% [25]. In the studied species, the average value of a Cyperaceae member was 57.54%, while the average of a Poaceae species was 60.31% (Figure 1). These results are comparable to those obtained by Gill et al. [26] on twelve barley varieties (overall mean of 64.8%) and Mashaly et al. [10] on some members of Cyperaceae (57.12–60.74%) and Poaceae (51.92–62.82%).

### 2.2. Mineral Content

**Figure 2:**
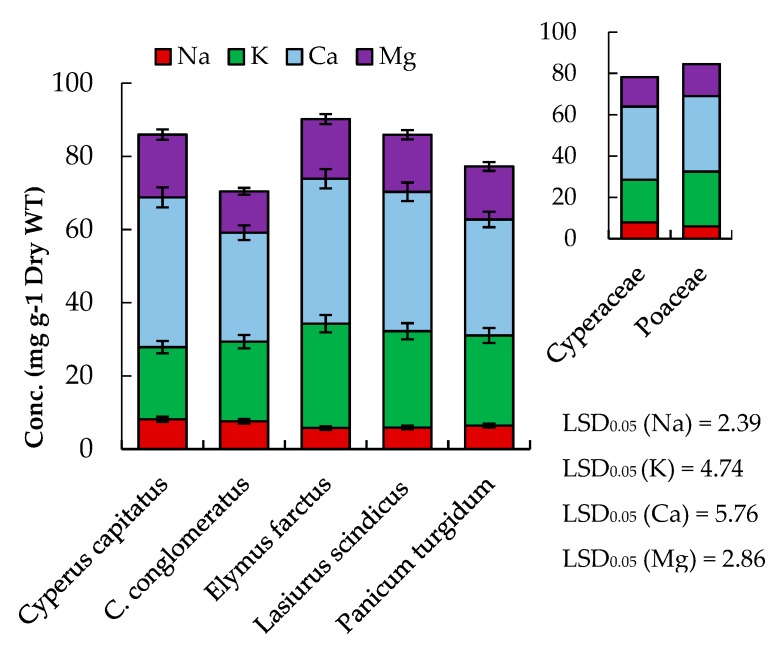
Macro-mineral composition of selected geophytes under study. Values are means (*n* = 3) with standard error bars. LSD_0.05_ is the least significant difference at the probability level of 0.05.

**Figure 3:**
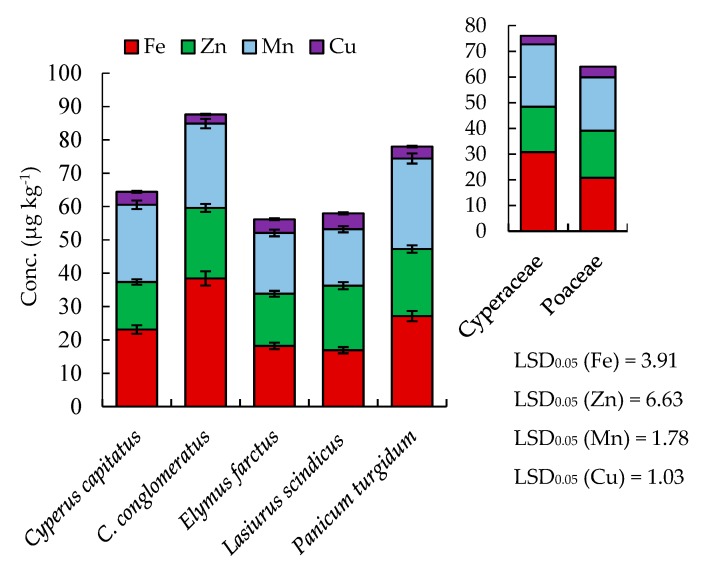
Micro-mineral composition of selected geophytes. Values are an average (*n* = 3) with standard error bars. LSD_0.05_ is the least significant difference at the probability level of 0.05.

Mineral composition has essential structural and physiological roles in animals as well as maintaining livestock health. In this study, the concentrations of the macro- (K, Ca, Mg, and Na) and micro-elements (Fe, Mn, Zn, and Cu) were estimated in the five studied geophytes (Figure 2 and Figure 3). According to the concentration in the studied geophytes, the macro-minerals can be sequenced as follows: Ca > K > Mg > Na, while the micro-minerals can be ordered as Fe > Mn > Zn > Cu. Moreover, different plant species had variable mineral concentrations. 

The mineral content of studied plant species ranged from 29.8–40.9 mg g^−1^ DW (Ca), 19.7–28.5 mg g^−1^ DW (K), 11.3–17.2 mg g^−1^ DW (Mg), 5.78–8.16 mg g^−1^ DW (Na), 16.93–38.44 µg g^−1^ (Fe), 14.20–21.14 µg g^−1^ (Zn), 16.93–27.16 µg g^−1^ (Mn), and 2.76–4.75 µg g^−1^ (Cu) (Figure 2 and Figure 3). Our results were similar to those reported by Abbasi et al. [27] on white clover and Vejnovic et al. [28] on natural grasslands. According to the Agricultural Research Council (ARC) [29] and the National Research Council (NRC) [30] systems, the requirement for mineral nutrients for gestating beef cows or lactating beef cows is 38.0 mg kg^−1^ for K, 15.4 mg kg^−1^ for Ca, 3.0 mg kg^−1^ for Mg, 6.8 mg kg^−1^ for Na, 45.0 µg kg^−1^ for Zn, 2.0 µg kg^−1^ for Mn, and 7.1 µg kg^−1^ for Cu. Thus the studied plant species of Poaceae and Cyperaceae have suitable contents of the minerals. In general, Cyperaceae members attained higher contents of Na, Fe, and Mn compared to Poaceae members, while the latter have higher contents of K (Figure 2 and Figure 3). 

### 2.3. Secondary Metabolites

The dry and saline habitats of the studied geophytes species are hypothesized as triggers for the synthesis of various secondary metabolites such as phenolics, flavonoids, alkaloids, saponins, and many other compounds that have protective and medicinal properties [31]. The present results showed that the contents of total phenolics, alkaloids, total flavonoids, saponins, and tannins were in the range of 26.34–9.59, 21.84–6.08, 18.27–5.31, 41.16–10.13, and 26.10–4.13 mg g^−1^ DW, respectively (Table 2). *Cyperus conglomeratus* attained the highest contents of all investigated secondary metabolites, where it attained about double-fold than *C. capitatus* regarding all secondary metabolites and more than double fold for the tested grasses (*E.* farctus, *L. scindicus*, and *P. turgidum*). These results are consistent with to those reported by Canty et al. [32] on *Lolium perenne*, and Kara and Sürmen [33] on rangeland and pasture plants of the genus of *Lotus, Astragalus,* and *Onobrychis*. It is worth mentioning here that *C. conglomeratus* has been reported to have several bioactive compounds such as flavonoids [25], glycosides, sterols, tannins, and terpenoids [34]. *Cyperus capitatus* has been reported to contained several bioactive constituents such as essential oil [35], quinones, and flavonoids [36]. 

Antinutrients or antinutritional factors are the compounds that act to reduce nutrient intake, digestion, absorption, and utilization, and also produce adverse effects. The major antinutrients are alkaloids, flavonoids, saponins, tannins, oxalates, cyanogenic glycosides, phytic acid, gossypol, goitrogens, lectins, chlorogenic acid, protease and amylase inhibitors. The content of antinutritional factors in the plant depends on secondary metabolites, which vary with plant species, phenological periods, and environmental conditions [37]. Excess content of saponins in the fodder adversely affects animals, causing a depression of growth rates, inhibiting enzyme activity, and leading to a reduction in nutrient absorption in the digestive tract, as well as that saponins are bitter-tasting molecules [38]. In addition, tannins are other compounds with high molecular weight and are soluble in water, when herbivorous grazed plants with a high content of tannins, they form a less digestive complex with dietary proteins and reduce the chances of growth and survival of animals [33]. Tannins are also characterized by an astringent taste, therefore, it is considered as antinutritional compounds [38]. Alkaloids are also commonly found in about 20% of vascular plant species, and when grazed by animals, they affect the nervous system and can cause paralysis and sudden deaths [39]. Overall, *C. conglomeratus* attained a high content of the secondary metabolites, which could be considered as an antinutritional compound. Therefore, this plant may be not suitable as an animal fodder although it has a high nutritive value. Some members of Cyperaceae are known to possess antifeedants compounds such as coumaran, remirol, cyperaquinone, furoquinones, and scabequinone [40].

### 2.4. Antioxidant Activity

**Figure 4:**
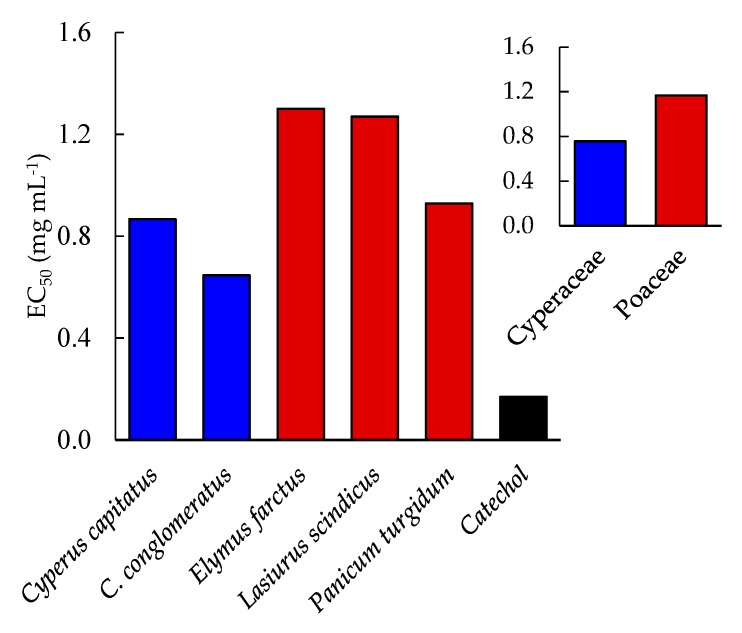
EC_50_ values of the different studied geophytes and catechol (standard).

The extracts of the five studied geophytes showed a significant increase in the scavenging activity of 2,2-diphenyl-1-picrylhydrazyl (DPPH) in a dose-dependent manner (Figure 4). At 1 mg mL^−1^, the extracts of *C. conglomeratus, E. farctus, P. turgidum, C. capitatus,* and *L. scindicus* showed scavenging activities of 63.87%, 54.26%, 52.31%, 51.52%, and 50.79%, respectively. Based on the EC_50_ values, the studied geophytes can be ordered as follows: *C. conglomeratus* > *C. capitatus* > *P. turgidum* > *L. scindicus* > *E. farctus*, where they attained the values of 0.65, 0.87, 0.93, 1.27, and 1.30 mg mL^−1^, respectively (Figure 4). However, the positive control or standard (catechol) attained an EC_50_ value of 0.17 mg mL^−1^. These data reflect that members of Cyperaceae are considered better candidates as antioxidant natural resources than the Poaceae members.

The antioxidant capacity of *C. conglomeratus* and *C. capitatus* can be attributed to the high content of bioactive compounds [9,41]. Moreover, *C. conglomeratus* is characterized by various biological activities such as anthelmintic, antidiarrheal, antidiabetic [41,42], antioxidant, antimicrobial [34,43], and cytotoxic activity [9].

On the other hand, *P. turgidum* has been reported to possess anti-inflammatory, antibacterial, antihepatotoxic, antifungal, and cytotoxic activities [44,45,46,47,48]. These biological activities were mainly due to steroidal saponins, which are characterized components of this genus [46]. In addition, this plant has other bioactive compounds such as phenolic acids (caffeic, ferulic, *p*-coumaric, protocatechuic, and *p*-hydroxybenzoic), and flavonoids (rutin and quercetin glycosides) [45].

## 3. Materials and Methods

### 3.1. Collection and Preparation of Plant Materials

**Figure 5:**
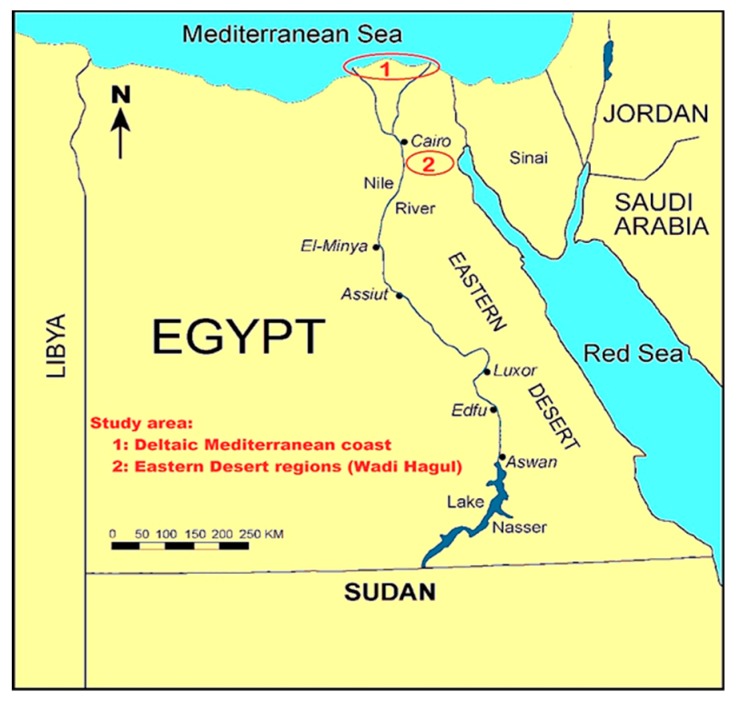
Map of Egypt showing the locations of plant sampling.

**Figure 6:**
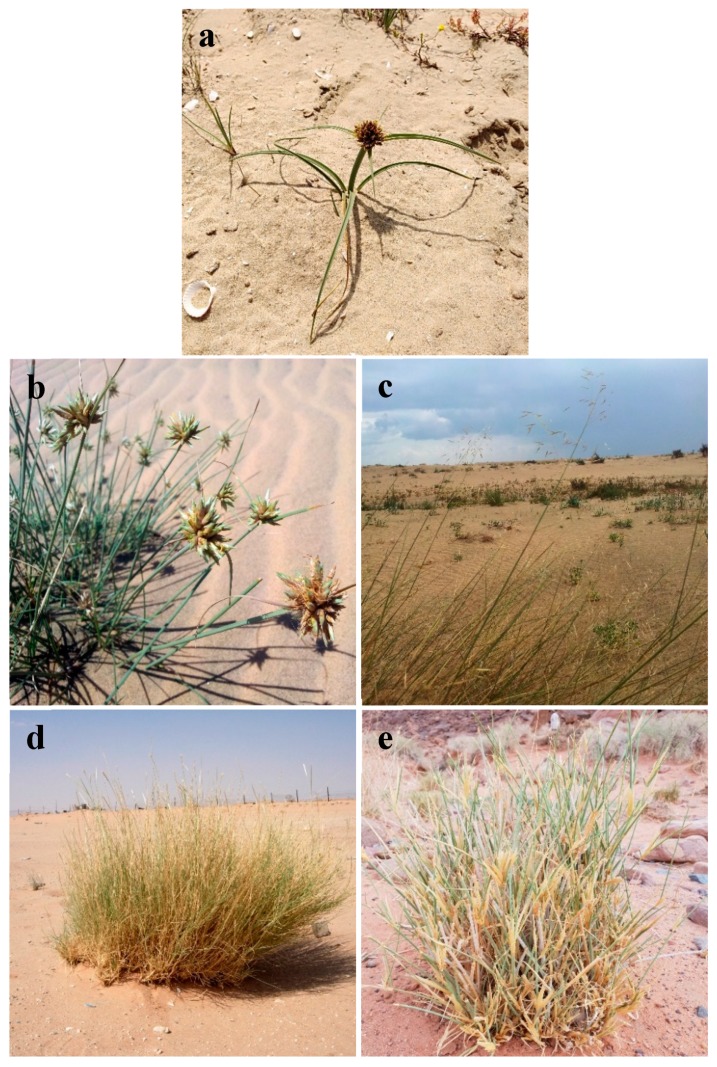
Close view of the five studied geophytes. (**a**) *Cyperus capitatus*, (**b**) *Cyperus conglomeratus*, (**c**) *Elymus farctus*, (**d**) *Lasiurus scindicus*, and (**e**) *Panicum turgidum*.

Five wild geophyte species were collected during the period of March 2018 from naturally growing populations distributed in different habitats of the northern part of the Mediterranean Sea (Nile Delta coast) and Eastern Desert regions (Wadi Hagoul) of Egypt (Figure 5). Two geophytes belong to Cyperaceae (*C. capitatus* and *C. conglomeratus*) and three belong to Poaceae (*E. farctus, L. scindicus,* and *P. turgidum*) (Table 3 and Figure 6). The plants were identified by Dr. Yasser El-Amier (author), and voucher specimens were deposited in the herbarium of the Faculty of Science at Mansoura University, Egypt. 

The collected aerial parts were handily cleaned and the died or yellow leaves were removed. The samples were washed three times by distilled water to remove dust and other residues, dried at room temperature (25 ± 2 °C) in a shaded place for several days till complete dryness and ground into powder. Finally, the dried samples were stored in paper bags until further analyses. 

### 3.2. Proximate Composition

Dry matter, moisture content, total ash content, fat, and fiber were determined in the collected aerial parts of the plants following the methods of AOAC [49]. Total nitrogen was determined by the micro-Kjeldahl method [50]. Protein contents of the plant species were determined by multiplying N contents by the factor 6.25 [49]. Glucose was determined according to the method of Feteris [51], while sucrose content was estimated following the method of Handel [52]. 

The total carbohydrate content for each sample was calculated by “difference”, in which the summation of the percentages of all the other proximate components was subtracted from 100 [49]. The potential energy (kcal 100 g^−1^ DW) was carried out using the Atwater general factors as described by FAO [53] by multiplying the values obtained for protein, carbohydrates, and fat by 4, 3.75, and 9, respectively; the results are expressed in kcal. The TDN was estimated according to the equation described by Abu-El-Naga and EL-Shazly [54] as follows:

TDN (%) = 0.623 (100 + 1.25 EE) − P 0.72(1)

where EE is the percentage of ether extract, and P is the percentage of crude protein.

### 3.3. Minerals Content Analysis

About 0.1 g of each air-dried powder of the plant aerial parts were digested by using concentrated HNO_3_ and gently heated until all the organic matter disappeared and the solution turned quite clear [55]. The clear samples were made up to a known volume using distilled water. Na and K were determined in all samples by a flame photometer (Jenway PFP7, Felsted, UK), while calcium, magnesium, copper, zinc, iron, and manganese were estimated using an atomic absorption spectrometer (A Perkin-Elmer, Model 2380, Waltham, MA, USA).

### 3.4. Phytochemical Analysis

The total phenolics content was determined according to the method of Sadasivam and Manickam [56]. In brief, a known weight (0.1 g) of dried plant tissue was ground in 80% (*v/v*) aqueous methanol. The mixture was filtrated via muslin and centrifugated at 10,000 rpm for 20 min. The supernatant was then evaporated, and the residue was redissolved in distilled water (5 mL). A mixture of 0.5 mL extract, 2.5 mL distilled water, 2 mL NaCO_3_ (20%), and 0.5 mL of Folin–Ciocalteu reagent was prepared and incubated for 40 min in a dark condition. Using a spectrophotometer (Spectronic 21D model, Milton Roy, CA, USA), the absorbance was measured at 725 nm. The content of total phenolics was determined based on a standard curve of gallic acid and expressed as mg gallic acid equivalent g^−1^ DW.

Tannins content was determined spectrophotometrically according to Sadasivam and Manickam [56]. One gram of plant tissue was extracted by 50 mL of methanol (80%, *v/v*) with continuous shaking for 24 h. The homogenate was centrifuged, and the supernatant was collected and raised to a known volume. About 5 mL of vanillin hydrochloride reagent (8% HCl in methanol and 4% vanillin in methanol 1:1 *v/v*) was added to 1 mL of the supernatant and incubated at room temperature for 20 min. The optical density was measured via spectrophotometer at 500 nm. The content of tannins was determined based on a standard curve of (0–100 μg) of tannic acid and expressed as mg g^−1^ DW. 

For the determination of saponins, 20 g of dried plant tissue was mixed with 100 mL of 80% (*v/v*) aqueous ethanol and heated over water bath at 55 °C for 4 h. The mixture was filtered, and the filtrate was collected, while the residue re-extracted with an additional 100 mL of 20% ethanol. The filtrates were combined and concentrated to reach 40 mL over a water bath. About 20 mL of diethyl ether was added to the concentrate in a separating funnel and shaken vigorously. The aqueous layer was heated over water bath till complete dryness and the dried residue was weighed as total saponins and expressed as mg g^−1^ DW [57]. 

Total flavonoid content was estimated by the AlCl_3_ colorimetric method [58]. In brief, an extract of 1 mg L^−1^ was prepared using methanol. Then, one mL (5%) of the previously prepared extract was mixed with 4 mL distilled water, 0.3 mL of NaNO_2_ (5%), and 0.3 mL of AlCl_3_. The mixture was left to stand for 6 min, then 2 mL of NaOH (1 M) was added, and the volume was rained to 10 mL by distilled water. The absorbance was measured after 15 min at 510 nm by a spectrophotometer (Spectronic 21D model, Milton Roy, CA, USA). A standard curve of rutin was prepared, and the amount of the total flavonoids was expressed as mg rutin equivalent g^−1^ DW. 

The alkaloids were extracted with 10% acetic acid in ethanol and determined based on the method of Harborne [59]. About 40 mL of 10% acetic acid in ethanol was added to 1 g of dried plant tissue in a beaker and allowed to stand for 4 h. The homogenate was filtered, and the filtrate was concentrated into one-quarter of the original volume over a water bath. The alkaloids were precipitated using concentrated ammonia solution. The residue was dried till constant weight, and the alkaloids were expressed as mg g^−1^ DW.

### 3.5. Determination of Antioxidant Activity

The antioxidant potential of the plant samples was evaluated based on the depletion of the color of the stable free radical DPPH, as described by [59]. In summary, equal volumes (2 mL) of both 0.15 mM DPPH and plant extracts in different concentrations (1.0, 0.8, 0.6, 0.4, 0.2, and 0.1 mg mL^−1^, in methanol) were mixed well in the test tubes. The reaction mixture was incubated in a dark condition at room temperature (25 °C) for 30 min. Immediately, the absorbance was measured by a spectrophotometer at 517 nm. A control treatment was achieved using methanol instead of plant extract. Antioxidant activity was expressed as

Scavenging activity (%) = [1 − (A _sample_/A _control_)] × 100 (2)

The EC_50_ (concentration of plant material that reduces the activity by 50%) was calculated graphically.

### 3.6. Statistical Analysis

The data in triplicates of proximate composition, minerals, secondary metabolites were subjected to a one-way ANOVA test after Duncan’s test to assess the variance between the five studied geophytes. The analysis was carried out using CoStat (version 6.311, CoHort Software, USA, www.cohort.com).

## 4. Conclusions

The present study revealed that geophytes *C. capitatus*, *C. conglomeratus*, *E. farctus, L. scindicus*, and *C. capitatus* have a considerable amount of the proximate composition as well as macro- and micro-minerals that enabling these plants to be potentially good candidates as forage plants, at least under the arid environments such as the Egyptian deserts. Based on the nutritive values, the geophytes in the present investigation can be ranked as follows: *Elymus farctus* > *C. conglomeratus* > *Lasiurus scindicus* > *Panicum turgidum > Cyperus capitatus*. However, *C. conglomeratus* attained high content of various secondary metabolites (total phenolics, tannins, total flavonoids, alkaloids, and saponins). On the other hand, the Cyperaceae members (*C. conglomeratus* and *C. capitatus*) showed significant antioxidant activity compared to the grasses. Therefore, we recommend further study to evaluate the anti-nutritional factors of the studied geophytes as well as to experiment on ruminant animals to evaluate their palatability and productivity. 

## Figures and Tables

**Table 1:** Proximate composition (on dry matter basis) of the studied geophytes collected from the Egyptian desert.

Proximate Composition	*Cyperus capitatus*	*Cyperus conglomeratus*	*Elymus farctus*	*Lasiurus scindicus*	*Panicum turgidum*	*p*-Value
Dry matter %	87.10 ± 4.94 ^a^	91.67 ± 3.65 ^a^	89.80 ± 5.09 ^a^	90.46 ± 3.12 ^a^	90.13 ± 5.11 ^a^	*0.58*
Moisture %	12.90 ± 1.29 ^a^	8.33 ± 0.83 ^a^	10.20 ± 1.02 ^a^	9.54 ± 0.95 ^a^	9.87 ± 0.99 ^a^	*0.51*
Ash %	10.75 ± 1.07 ^a^	10.54 ± 1.05 ^a^	9.39 ± 0.94 ^a^	9.07 ± 0.91 ^a^	8.62 ± 0.86 ^a^	*0.47*
Fiber %	12.51 ± 1.25 ^a^	11.97 ± 0.98 ^a^	13.40 ± 1.34 ^a^	12.89 ± 1.29 ^a^	13.84 ± 0.88 ^a^	*0.23*
Fat %	3.89 ± 0.28 ^cd^	3.19 ± 0.23 ^d^	5.13 ± 0.38 ^ab^	4.61 ± 0.34 ^bc^	5.87 ± 0.43 ^a^	*0.0013*
Protein %	16.38 ± 1.20 ^ab^	17.13 ± 1.25 ^a^	13.50 ± 0.99 ^cd^	14.88 ± 1.09 ^bc^	12.06 ± 0.88 ^d^	*0.0004*
Sucrose %	1.89 ± 0.14 ^a^	1.98 ± 0.15 ^a^	1.74 ± 0.13 ^a^	1.81 ± 0.13 ^a^	1.52 ± 0.11 ^a^	*0.98*
Glucose %	0.72 ± 0.05 ^a^	0.94 ± 0.07 ^a^	0.78 ± 0.06 ^a^	0.82 ± 0.06 ^a^	0.68 ± 0.05 ^a^	*0.78*

Values are mean of triplicates ± standard error. Different letters within each measurement mean values of significant variation at *p* ≤ 0.05.

**Table 2:** Secondary compounds (mg g^−1^ DW) of the studied geophytes collected from the Egyptian desert.

Secondary Compounds	*Cyperus capitatus*	*Cyperus conglomeratus*	*Elymus farctus*	*Lasiurus scindicus*	*Panicum turgidum*
Total phenolics	16.02 ± 0.86 ^b^	26.34 ± 1.41 ^a^	13.57 ± 0.73 ^bc^	9.59 ± 0.51 ^d^	10.69 ± 0.57 ^cd^
Alkaloids	15.15 ± 0.81 ^b^	21.84 ± 1.17 ^a^	16.10 ± 0.86 ^b^	6.89 ± 0.37 ^c^	6.08 ± 0.33 ^c^
Total flavonoid	10.57 ± 0.57 ^b^	18.27 ± 0.98 ^a^	8.02 ± 0.43 ^c^	5.31 ± 0.28 ^d^	5.82 ± 0.31 ^d^
Saponins	19.59 ± 1.05 ^b^	41.16 ± 2.21 ^a^	18.60 ± 1.00 ^c^	10.13 ± 0.54 ^d^	11.41 ± 0.61 ^d^
Tannins	12.42 ± 0.67 ^b^	26.10 ± 1.40 ^a^	12.80 ± 0.69 ^b^	5.46 ± 0.29 ^c^	4.13 ± 0.22 ^c^

Values are mean of triplicates ± standard error. Different letters within each measurement mean values of significant variation at *p* ≤ 0.05.

**Table 3:** Plant species name, family, duration, and chorotype of studied wild geophytes naturally growing in the Egyptian desert.

Botanical Name	Family	Common Name	Duration	Chorotype	Voucher Code
*Cyperus capitatus* Vand.	Cyperaceae	Seed	Perennial	ME	Mans.030303012
*Cyperus conglomeratus* Rottb.	Cyperaceae	Seed, Oshb	Perennial	SA-SI + S-Z	Mans.030303008
*Elymus farctus* (Viv.) Runem. ex Melderis	Poaceae	Gazzoof	Perennial	ME	Mans.160506018
*Lasiurus scindicus* Henrard.	Poaceae	Sammat	Perennial	SA-SI + S-Z	Mans.161219003
*Panicum turgidum* Forssk.	Poaceae	Thommam, Shoosh	Perennial	SA-SI	Mans.161620007

ME: Mediterranean; SA-SI: Saharo-Sindian; S-Z: Sudano-Zambezian.
